# Persumed sympathetic Ophthalmia after scleral buckling surgery: case report

**DOI:** 10.1186/s12348-020-00233-z

**Published:** 2021-02-22

**Authors:** Seyedeh Maryam Hosseini, Nasser Shoeibi, Mahdieh Azimi Zadeh, Mahdi Ghasemi, Mojtaba Abrishami

**Affiliations:** Eye Research Center, Khatam-al-Anbia Eye Hospital, Mashhad University of Medical Sciences, Qarani Boulevard, Mashhad, 9195965919 Iran

**Keywords:** Sympathetic ophthalmia, Scleral buckling, Uveitis, Cryoretinopexy, Case report

## Abstract

**Background:**

Scleral buckling (SB) is usually considered an extraocular operation premeditated to have a low risk of sympathetic ophthalmia (SO). Here we report a rare case of presumed SO in a young female patient following SB.

**Case presentation:**

A nineteen-year-old female patient was referred for visual loss in her left eye due to macula off inferior long-standing rhegmatogenous retinal detachment (RRD). The best corrected visual acuity (BCVA) was 20/400 in the left eye. SB with 360 degrees encircling band, an inferior segmental tire with one spot cryoretinopexy at the break site, and subretinal fluid drainage was performed. BCVA was improved to 20/80 and the retina was totally attached 1 week after the operation. The patient referred to the hospital 6 weeks later with severe visual loss in both eyes as counting finger 1 m. Patient examination indicated bilateral multifocal serous retinal detachment (SRD) and vitreous cells. The patient, diagnosed with SO, received intravenous corticosteroid pulse therapy and mycophenolate mofetil for treatment. The inflammation was controlled and SRD resolved after a 5-day intravenous treatment without being relapsed after 6 months. Consequently, BCVA became 20/20 and 20/50 in the right and left eye, respectively, after 6 months. The findings of systemic workup were negative for any extraocular disease or systemic involvement.

**Conclusion:**

Since SB is a procedure without manipulating intraocular tissues, it is considered to impose a low risk for SO. This report presented SO occurrence after successful SB. Some factors may induce SO, including inciting the choroid and retinal pigment epithelium with cryoretinopexy or perforating for drainage.

## Introduction

Sympathetic ophthalmia (SO) is a rare bilateral diffuse granulomatous panuveitis that may occur after surgery or trauma to one eye, as penetrating injury and exposure of the uvea [1]. The incidence of SO is reported ranging 0.01–0.5% after intraocular surgery [1–5]. Surgeries with manipulation and irritation of the choroid and retina are considered as risk factors [1]. The interval between the ocular injury and the onset of SO varies greatly, from a few days to decades, with most of the cases occurring within 3 months after the injury to the exciting eye and 90% during the first year [2–4]. The inciting ocular surgery varies, including cataract extraction, secondary intraocular lens placement, trabeculectomy, vitrectomy, cyclodestruction, iridectomy, and evisceration [1]. Recently, there has been an increasing trend of SO incidence after intraocular and vitreoretinal surgeries [6].

Scleral buckling (SB) is usually considered an extraocular operation, a privilege over vitrectomy, and SO is presumed to have a very low risk after SB. Here we report an unusual rare case of SO following successful SB surgery which was combined with subretinal fluid drainage (SRFD) and cryoretinopexy.

## Case presentation

A nineteen-year-old female was referred for visual loss and superior visual field defect in her left eye (LE). Best corrected visual acuity (BCVA) was 20/20 and 20/400 in the right eye (RE) in the LE, respectively. The anterior segment examination was within normal limits except for the detection of Shaffer sign in the LE. The results of the fundus examination revealed macula off inferior long-standing rhegmatogenous retinal detachment (RRD), 3 h of proliferative vitreoretinopathy (PVR), retinal break, and lattice degeneration. The patient had no history of head or ocular trauma or any intraocular surgery. SB with 360 degrees encircling silicone band (silicone band type 240, FCI Inc., Paris, France) and inferior segmental silicone tire (asymmetrical silicone tire type 276, FCI Inc., Paris, France) with one spot cryoretinopexy at the break site, and SRFD due to chronicity of the RRD was performed. During this process, SRFD was not inadvertent. Scleral thinning was performed after passing the band and tire. Moreover, the needle of polyester spatula suture (Mersilene, Ethicon LLC, Johnson and Johnson Inc., USA) was used to drain the fluid after the cauterization of the choroid. BCVA was improved to 20/80 and the retina was totally attached 1 week after the operation. The patient was followed up receiving a topical antibiotic (Chlobiotic, Sina Darou, Tehran, Iran) and topical corticosteroid (Bethasonate, Sina Darou, Tehran, Iran) for 3 weeks.

**Fig. 1:**
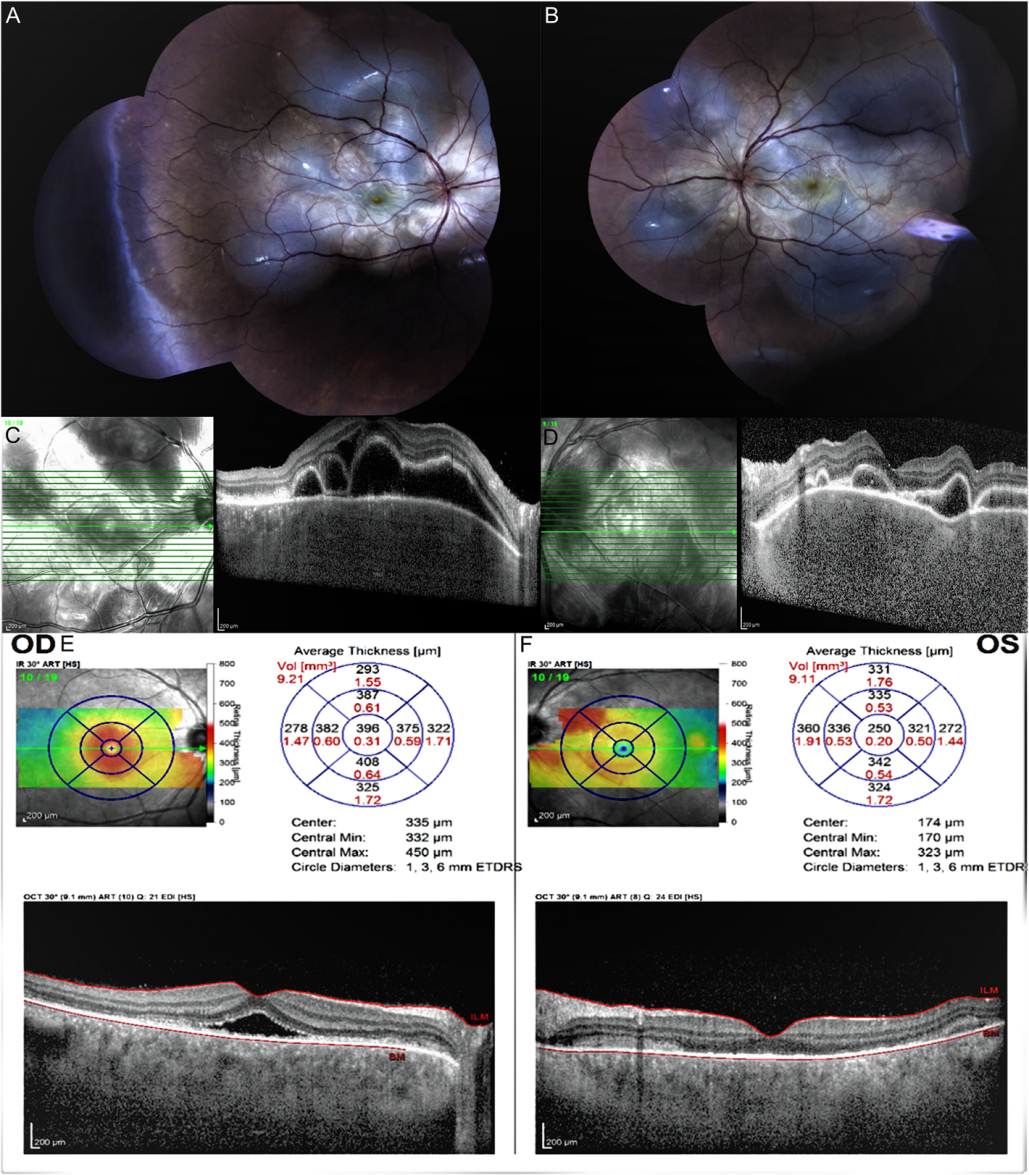
Fundus photography of the right (**a**) and the left (**b**) eye reveals bilateral disc swelling and multifocal serous retinal detachment at the posterior pole. Enhanced depth imaging optical coherent tomography (EDI-OCT) of the right (**c**) and the left (**d**) eye at the first presentation after the development of sympathetic ophthalmia (SO) discloses multi-lobular serous retinal detachment, septate subretinal spaces, hyperreflective dots in subretinal fluid, choroidal thickening, and undulation of the retinal pigment epithelium. The results of EDI-OCT of the right (**e**) and left (**f**) eye shows the significant response with resolution of serous retinal detachment and decreased choroidal thickening 5 days after pulse corticosteroid therapy

**Fig. 2:**
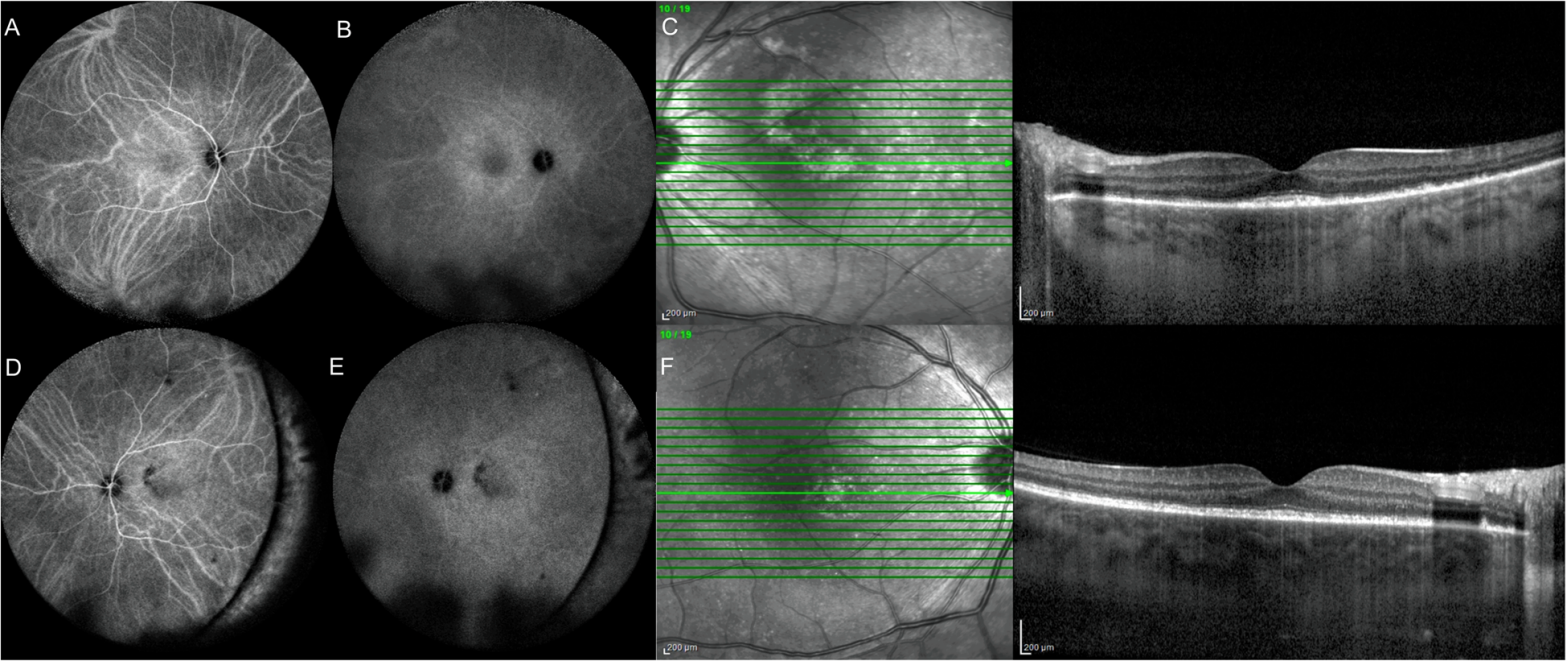
Indocyanine green angiography of the right (**a**, **b**) and the left eye (**d**, **e**) at the third month disclosed only a few hypofluorescent dark dots (HDD) in both eyes and mild background hyperflorescence at the posterior pole indicating a response to treatment with mild subclinical inflammation of choroidal stroma. Enhanced depth imaging optical coherent tomography of the right (**c**) and left (**f**) eyes at 3 months follow-up showed complete response with resolution of serous retinal detachment and decreased choroidal thickening and inflammation

The patient referred to the hospital with severe visual loss in both eyes 6 weeks later. On examination, BCVA of both eyes was counting finger at one meter and the anterior segment was within normal limits, except 1+ anterior chamber and anterior vitreous cells in both eyes. Bilateral multifocal serous retinal detachment (SRD) was obvious in funduscopy (Fig. 1a, b). The results of enhanced depth imaging optical coherence tomography (EDI-OCT) (Spectralis HRA +OCT, Heidelberg Engineering, Heidelberg, Germany) revealed multilobular SRD, septated subretinal spaces, hyperreflective dot reflexes in subretinal fluid, choroidal thickening, and retinal pigment epithelium undulation (Fig. 1c, d). Chorioscleral junction was not detected due to diffuse severe choroidal thickening. According to the results of examination and imaging, as well as the absence of any other ocular trauma history except for SB surgery, the diagnosis of SO was presumed incited by SB surgery. Therefore, the patient was admitted and received high-dose (1 g/day) intravenous methylprednisolone treatment for 5 days. This five-day pulse therapy led to the control of inflammation and the significant resolution of SRD (Fig. 1e, f). The patient underwent all systemic work-ups for common causes of choroiditis, including sarcoidosis, tuberculosis, syphilis, and autoimmunity markers were inconclusive. Moreover, familial, drug, and past medical history were unremarkable. After pulse therapy, oral prednisolone (50 mg/day) and mycophenolate mofetil (MMF) (2 g/day) were initiated. On the fourth day, fluorescein angiography showed no significant dye pooling except for mild leakage; also, it revealed mixed hypo- and hyper-fluorescent dots scattered at the posterior poles presumably due to choroidal hypoperfusion and abnormal leakage. Since indocyanine green angiography (ICGA) was not available at that time, it was not performed at the first presentation of SO. Prednisolone tapered rapidly due to the increased blood glucose, and subtenon triamcinolone acetonide (TriamHEXAL, Hexal AG, Holzkirchen, Germany) (40 mg/1 cc) was injected bilaterally. Induced diabetes was controlled with oral medication which was stopped after 1 month. Prednisolone was reached to 5 mg/d at the end of the second month with continuing MMF (2 g/d). The blood glucose was checked frequently, which was within normal limits during the follow-up and did not relapse during 6-months follow-ups. Following one-year treatment with MMF 2 g/d and prednisolone 5 mg/d, inflammation was reduced considerably and the retina was attached completely in both eyes. Final BCVA was obtained as 20/20 and 20/50 in the RE and LE, respectively. After 3 months of immunosuppressive treatment, ICGA disclosed only a few hypofluorescent dark dots in both eyes and mild background hyperflorescence at the posterior pole indicating a response to treatment with mild subclinical inflammation of choroidal stroma (Fig. 2).

## Discussion

Here, we report a rare case of SO following uncomplicated SB surgery accompanied by cryoretinopexy and SRFD. In this regard, it is recommended that SO be taken into consideration in any case of bilateral panuveitis associated with multiple SRD and a history of penetrating ocular surgery.

In several studies, the occurrence of SO has been reported after vitreoretinal surgery [2, 4–6]. It is postulated that trauma to the uveal tract in the context of inadvertent perforation, SRFD, or cryotherapy during SB surgery might be related to deliberate uveal antigens, melanin or outer photoreceptor antigens resulting to access to the lymphatic systems of the conjunctival tissue. This process may associated to exciting of delayed hypersensitivity reaction inside the eye [4–6]. The mechanism of hypersensitivity in the sympathizing eye may be due to the exposure of the uveal tissue to the conjunctival lymphatic system leading to a cell-mediated immune response [6]. This mechanism, owing to more uveal incarceration, can explain the relationship between an increase in the trend of transconjunctival sutureless vitrectomy application and a growth in the incidence of SO following vitreoretinal surgery [6]. In our case, SRFD seemingly increased the risk of uveal exposure to conjunctival lymphatic tissue. In a study conducted by Kilmartin et al., it was reported that RRD surgery was the most common procedure associated with the development of SO, with the risk of SO after vitrectomy being as twice as that of external scleral buckling, without any gender predilection [7].

The onset of SO symptoms after the operation usually occurs between 3 weeks and 6 months after surgery due to delayed hypersensitivity [8]. Regarding the subject in this study, SO was developed 6 weeks later. In a case series study carried out by Ozbek et al. [4], three cases of SO incidence occurred following SB; however, two patients had combined vitrectomy with SB. In the aforementioned research, SO occurred only in one case after encircling buckle combined with 360 indirect retinal photocoagulations and SRFD, in contrast to our case that had only one cryoretinopexy spot and one drainage site. In another similar case report by Parvaresh and Falavarjani, SO was found in a case with a history of SB revision after 4 years. In this case, the second surgery was combined with SRFD and cryoretinopexy [9]. Nonetheless, in our case, SO happened in the first operation without any surgical history.

In a recent study performed by Tyagi et al., the incidence of SO following vitreoretinal surgery was estimated at 0.038% of all vitrectomy cases, and vice versa, 9% of all cases of SO had vitreoretinal surgery [6]. In this research, 75% of cases underwent multiple ocular surgeries before the development of SO [6]. Furthermore, the most common anterior segment was non-granulomatous uveitis in 50% of subjects, in contrast to SRD occurring in 62.5% of cases [6]. In our case, it was found that anterior segment inflammation was less severe than the posterior segment chorioretinal findings.

Most of the cases presented in studies [4–9] had simultaneous SB and vitrectomy, previous trauma, or multiple surgeries. Nevertheless, the subject of our report had no extensive retinal or uveal tissue manipulation and no combined surgery or previous surgery, highlighting the importance of this report. One of the underlying reasons for this susceptibility might be related to the Asian ethnicity and higher prevalence of Vogt-Koyanagi-Harada (VKH) in this region.

One of the most important differential diagnoses of our case is related to VKH which is a common uveitis etiology with similar clinical and imaging findings with SO [1, 10]. Although sympathizing eye in SO presents clinically with nongranulomatous uveitis at first, it progresses to granulomatous uveitis afterward [8]. shows three successive stages: posterior uveitis, anterior segment involvement associated with posterior uveitis, and finally anterior granulomatous uveitis. Both anterior and posterior uveitis are present in SO patients within 2 weeks of disease onset [8]. Generally, the main differentiating clue between SO and VKH is the history of prior surgery or trauma in SO. Considering this, in our case, clinical features were more similar to SO than to VKH, as our patient had no systemic sign or symptom attributed to VKH or other systemic diseases causing choroiditis.

The results of SO imaging, based on FAG, revealed multiple hyperfluorescent pinpoints leakages associated with late pooling resembling VKH and hypofluorescent foci during the early phase of angiography. Moreover, the late phase of FAG showed hyperfluorescence similar to acute posterior multifocal placoid pigment epitheliopathy [10]. In our case, apparently due to the performance of FAG 4 days after the steroid therapy, hyperfluorscence was less prominent than hypofluorescence that was compatible with the location of granuloma and cellular infiltration. The most common features of ICGA imaging are multiple hypocyanescent spots. In the acute phase of SO, EDI-OCT discloses multiple SRD associated with hyperreflective septa, massive choroidal thickening, and loss of normal choroidal vascular architectures, as well as irregular photoreceptor outer segments similar to what was observed in the acute phase of VKH. The visual outcome is worse in SO as compared with VKH disease [8]. Moreover, BCVA was improved in our case due to early diagnosis and prompt, aggressive, and adequate treatment.

In conclusion, the diagnosis of sympathetic ophthalmia should be taken into account in any case of bilateral uveitis or bilateral SRD following scleral buckling with uveal tract violation, such as cryopexy or SRFD.

## Data Availability

The datasets used and/or analyzed during the current study can be provided by the corresponding author on reasonable request.

## Abbreviations

BCVA: Best corrected visual acuity
EDI-OCT: Enhanced depth imaging optical coherent tomography
OCT: Optical coherent tomography
FAG: Fluorescein angiography
ICGA: Indocyanine green angiography
RE: Right eye
LE: Left eye
SRD: Serous retinal detachment
RRD: Rhegmatogenous retinal detachment
RPE: Retinal pigment epithelium
SB: Scleral buckling
SRFD: Subretinal fluid drainage
SO: Sympathetic ophthalmia
